# Brentuximab‐Vedotin als Mono‐ oder Kombinationstherapie bei kutanen peripheren T‐Zell‐Lymphomen – eine retrospektive Analyse

**DOI:** 10.1111/ddg.15826_g

**Published:** 2025-11-14

**Authors:** Caroline Glatzel, Inga Hansen‐Abeck, Nina Booken, Johannes Düll, Matthias Goebeler, Patrick Schummer, Marion Wobser

**Affiliations:** ^1^ Klinik und Poliklinik für Dermatologie Venerologie und Allergologie Universitätsklinikum Würzburg; ^2^ Klinik und Poliklinik für Dermatologie und Venerologie Universitätsklinikum Hamburg‐Eppendorf, Hamburg; ^3^ Medizinische Klinik und Poliklinik II Universitätsklinikum Würzburg

**Keywords:** Brentuximab‐Vedotin, Brentuximab‐Vedotin Mono‐/Kombinationstherapie, Kutane periphere T‐Zell‐Lymphome (PTCL), kutane CD30‐positive periphere T‐Zell‐Lymphome, brentuximab vedotin, brentuximab vedotin mono‐ and combination therapy, cutaneous CD30‐positive peripheral T cell lymphomas, Cutaneous peripheral T cell lymphomas (PTCL)

## Abstract

**Hintergrund:**

Kutane periphere T‐Zell‐Lymphome (PTCL) sind selten und zeigen einen aggressiven Verlauf mit limitiertem Therapieansprechen. Die Wirksamkeit von Brentuximab‐Vedotin bei kutanen PTCL wurde bisher nicht systematisch untersucht.

**Patienten und Methodik:**

In dieser retrospektiven Datenanalyse evaluierten wir die Therapie mit Brentuximab‐Vedotin als Mono‐ oder Kombinationstherapie bei kutanen CD30‐positiven PTCL (n  =  9).

**Ergebnisse:**

Insgesamt zeigten sich gutes Ansprechen und akzeptable Verträglichkeit der Therapie. Ein Patient erzielte eine nahezu komplette Remission, bei fünf Patienten wurde eine partielle Remission und bei zwei Patienten ein gemischtes Ansprechen als bestes Ansprechen (*best overall response*, BOR) beobachtet. Im Median vergingen 31,5 Tage (Interquartilabstand 12–53) bis zum Therapieansprechen. Die mediane Ansprechdauer war mit 4,3 Monaten kurz; das mediane Gesamtüberleben seit Einleitung der Brentuximab‐Vedotin‐Therapie betrug 15,2 Monate. Vier von neun Patienten verstarben an ihrer fortgeschrittenen Lymphomerkrankung, ein Patient verstarb aus anderer Ursache.

**Schlussfolgerungen:**

Brentuximab‐Vedotin als Mono‐ oder Kombinationstherapie ist bei Patienten mit kutanem peripheren T‐Zell‐Lymphom eine rasch wirksame und verträgliche Therapieoption bei allerdings nur kurzer Ansprechdauer.

## EINLEITUNG

Die Gruppe der kutanen peripheren T‐Zell‐Lymphome (*cutaneous peripheral T cell lymphomas*, PTCL) stellt eine sehr seltene Form primär kutaner Lymphome dar, die häufig einen aggressiven Verlauf mit meist nur kurzfristigem Therapieansprechen zeigt.^1^ In der Regel wird eine Polychemotherapie, meist nach dem CHOP‐Schema (Cyclophosphamid, Doxorubicin, Vincristin, Predniso[lo]n), eingesetzt.^2^, ^3^

Seit dem Jahr 2017 ist die Systemtherapie mit Brentuximab‐Vedotin (BV)^4^, ^5^ bei CD30‐positiven kutanen T‐Zell‐Lymphomen zur Zweitlinientherapie zugelassen und zeigt bei der Mycosis fungoides (MF), dem Sézary‐Syndrom und den CD30‐positiven Lymphoproliferationen eine hohe Effektivität bei akzeptabler Verträglichkeit.^6^, ^7^

In der zulassungsrelevanten ALCANZA‐Studie (NCT01578499) erwies sich die Systemtherapie mit BV im Vergleich zur Kontrollgruppe (Systemtherapie mit Methotrexat oder Bexaroten) in entscheidenden Endpunkten als deutlich überlegen. Ein über mindestens 4 Monate anhaltendes objektives Therapieansprechen (ORR4) wurde bei 54,7% der Patienten im BV‐Arm beobachtet, gegenüber 12,5% in der Kontrollgruppe.^8^ Eine komplette Remission (CR) erzielten 17,2% der BV‐Patienten, im Vergleichsarm waren es 1,6%. Das mediane progressionsfreie Überleben (PFS) im BV‐Arm betrug 16,7 (95%‐Konfidenzintervall [KI] 15,4–21,6) Monaten im Vergleich zu 3,5 (95%‐KI 2,4–4,6) Monaten in der Kontrollgruppe. Die Zeit bis zur nächsten Tumortherapie (TTNT; *time to next treatment*) war mit 14,2 Monaten deutlich länger als die 5,6 Monate in der Kontrollgruppe.^7^ Patienten mit kutanem PTCL wurden nicht in die ALCANZA‐Studie eingeschlossen, gleichwohl liegen Daten zur CD30‐Expression auch für diese Lymphomentität vor.^9^

Obwohl BV prinzipiell auch für kutane CD30‐positive PTCL zugelassen ist, wurde die Wirksamkeit von BV als Mono‐ oder Kombinationstherapie bei kutanen PTCL bisher nicht systematisch untersucht. Wir stellen daher klinische Daten von neun Patienten mit CD30‐positiven kutanen PTCL aus zwei Zentren (Hamburg, Würzburg) vor, die eine Mono‐ oder Kombinationstherapie mit BV erhielten.

## PATIENTEN UND METHODIK

In dieser retrospektiven Analyse wurden das Behandlungsschema, die Verträglichkeit und Effektivität einer Therapie mit BV als Mono‐ oder Kombinationstherapie bei Patienten mit CD30‐positiven PTCL untersucht (n  =  9). Die entsprechenden Daten zu Diagnose, Vor‐/Folgetherapien, Therapieschema, Ansprechen und unerwünschten Ereignissen wurden aus den elektronischen Krankenakten der beiden teilnehmenden Zentren (Hamburg, Würzburg) extrahiert.

Das Ansprechen auf die Therapie war definiert als: *(1)* Komplettremission (*complete response*, CR) bei vollständiger Abheilung aller Tumorparameter des kutanen PTCL; nahezu Komplettremission (nearCR) bei vollständiger Abheilung aller klinischen bekannten Tumorparameter des kutanen PTCL mit Ausnahme singulärer/lokalisierter kutaner Läsionen; *(2)* partielle Remission (PR) als Rückgang aller messbaren Tumorparameter um ≥ 30% ohne Anzeichen einer fortschreitenden Erkrankung; *(3)* gemischtes Ansprechen (*mixed response*, MR) bei gleichzeitiger Regression und Progression der sicht‐ und messbaren Tumorparameter; *(4)* stabile Erkrankung (SD), wenn weder CR/PR noch Progress in allen von der Krankheit betroffenen Läsionen erreicht wurden; und *(5)* fortschreitende Krankheit (PD; *progressive disease*) bei Größenzunahme der bekannten Läsion um ≥ 20% oder Tumorprogression in einem Organsystem (zum Beispiel kutane oder nodale/viszerale Manifestationen).

Das beste Gesamtansprechen (BOR) wurde als bestes Ansprechen nach Einleitung von BV definiert. Das progressionsfreie Überleben (*progression‐free survival*, PFS) wurde als Zeitraum von Beginn der BV‐Therapie bis zum Fortschreiten der Krankheit definiert und nach der Kaplan‐Meier‐Methode berechnet. Die Zensierung erfolgte bei Tod oder letzter Nachuntersuchung. Der Median der Nachbeobachtungszeit wurde mit dem invertierten Kaplan‐Meier‐Ansatz berechnet (Datenstichtag 30. Juni 2024). Sämtliche Datenanalysen erfolgten mit R (Version 4.2.1; R‐Pakete: survival, survminer, ggplot2, ranger, prodlim).

## ERGEBNISSE

In diese retrospektive Datenanalyse wurden neun Patienten mit CD30‐positiven kutanen PTCL aus zwei Hauttumorzentren (Würzburg, Hamburg) eingeschlossen. Sieben Patienten waren an einem PTCL ohne weitere Spezifizierung (PTCL‐NOS) erkrankt, zwei an einem kutanen follikulären T‐Helferzell‐Lymphom (FTHZL). Das Erkrankungsstadium war in der Mehrheit der Fälle fortgeschritten mit generalisierter Hautbeteiligung (Stadium T3 nach EORTC/ISCL)^10^. Alle Patienten hatten zuvor mindestens eine Systemtherapie erhalten. In den teilnehmenden Hauttumorzentren gab es keinen Patienten, der aufgrund seiner Komorbidität von einer Behandlung mit BV ausgeschlossen wurde. Zu Beginn der BV‐Therapie waren die Patienten im Median 53 Jahre alt (Bereich: 31–77 Jahre). Die immunhistochemisch erfasste CD30‐Expression der kutanen Manifestationen war variabel (≤ 10% bei 6 Patienten, 30% bei 2 Patienten und 60% bei 1 Patienten) (Tabelle 1).

**TABELLE 1:** Patientencharakteristika.

**Patienten‐ID**	**Geschlecht**	**Alter bei Einleitung BV**	**CD30‐Expression**	**Tumorstadium**	**Zeitintervall zwischen ED und Start BV In Monaten**	**Systemtherapien vor Einleitung von BV (in zeitlicher Abfolge)**
1	Weiblich	77	< 5%	T3bN2M0	94	Methotrexat, Bexaroten
2	Männlich	67	10%	T3bN2M0	13	CHOP
3	Weiblich	53	5%	T3bN0M0	92	Bexaroten, Methotrexat, CHOP, Bexaroten + orale PUVA, Gemcitabin
4	Männlich	52	5%	T3bN1M0	19	Bexaroten
5	Männlich	50	60%	T3bN0M0	8	Methotrexat
6	Männlich	51	≤ 5%	T3bN0M0	71	Methotrexat, Gemcitabin
7	Männlich	68	≤ 5%	T3bN0M0	216	CHOP, DHAP, Methotrexat
8	Männlich	65	30%	T3bN2M0	5	Hydroxyurea
9	Weiblich	31	30%	T3N3M0	40	Methotrexat, Gemcitabin, CHOP

*Abk*.: BV, Brentuximab‐Vedotin; CHOP, Cyclophosphamid, Doxorubicin, Vincristin, Predniso(lo)n; PUVA, Psoralen + UV‐A; DHAP, Dexamethason, Cytarabin, Cisplatin

Die Gabe von BV erfolgte entweder als Monotherapie (3/9), simultan mit einer lokalisierten Radiatio (4/9) oder in Kombination mit Cyclophosphamid, Doxorubicin und Prednisolon (CHP) (2/9). Im Median erhielten die Patienten fünf Zyklen BV (Bereich: 3–13), mindestens jedoch drei Gaben. Unter diesen Therapiemodalitäten erzielte BV eine gute Effektivität (nearCR in 1/9, PR in 5/9, MR in 2/9 Patienten, PD in 1/9 [primäres Therapieversagen]). Patienten mit hohen CD30‐Expressionsraten (30% [n  =  2], > 60% [n  =  1]) zeigten kein besseres Ansprechen auf die BV‐Therapie als Patienten mit deutlich niedrigeren CD30‐Expressionswerten (Tabellen 1, 2).

**TABELLE 2:** Therapie und Ansprechen. Das Ansprechen auf die Therapie war definiert als: (a) Komplettremission (CR) bei vollständiger Abheilung aller klinischen Tumorparameter; nahezu CR (nearCR) bei Abheilung aller Tumorparameter bis auf eine kleine Läsion; (b) partielle Remission (PR) bei Rückgang der Tumorparameter um ≥ 30% ohne Fortschreiten; (c) gemischtes Ansprechen (mixed response, MR) bei gleichzeitiger Regression und Progression; (d) stabile Erkrankung (SD) bei keinem CR/PR oder Progression; (e) fortschreitende Krankheit (PD) bei ≥ 20% Größenzunahme oder Tumorprogression in einem Organsystem.

**Patienten‐ID**	**BV‐Mono‐/Kombinationstherapie**	**Bestes Gesamtansprechen**	**Regression der Läsionen im Vergleich zu Start BV**	**Folgetherapie (in zeitlicher Abfolge)**	**Status bei der letzten Kontrolle**
1	BV + RTX (30 Gy)	nearCR	99%	Bexaroten	Am Lymphom verstorben
2	BV	PR	≥ 30%	NA	Verloren für die Nachbeobachtung
3	BV	MR	≥ 30%	Keine	Am Lymphom verstorben
4	BV	PD	Keine	NA	Verloren für die Nachbeobachtung
5	BV + RTX (6 Gy)	PR	≥ 30%	CHOP	Am Lymphom verstorben
6	BV + CHP	PR	30%	Keine	Am Lymphom verstorben
7	BV + CHP	MR	30%	Mogamulizumab + TSEBT	Am Leben
8	BV + RTX (30 Gy)	PR	50%	Keine	An anderer Ursache gestorben
9	BV + RTX (22 Gy)	PR	75%	Allogene Stammzelltransplantation	Am Leben

*Abk*.: BV, Brentuximab‐Vedotin; RTX, Radiatio; CHP, Cyclophosphamid, Doxorubicin, Predniso(lo)n; nearCR, nahezu komplette Remission; PR, partielle Remission; PD, progressive disease; MR, gemischtes Ansprechen; NA, nicht angegeben; CHOP, Cyclophosphamid, Doxorubicin, Vincristin, Predniso(lo)n, TSEBT, *total skin electron beam radiation* (Ganzhautbestrahlung)

**ABBILDUNG 1:**
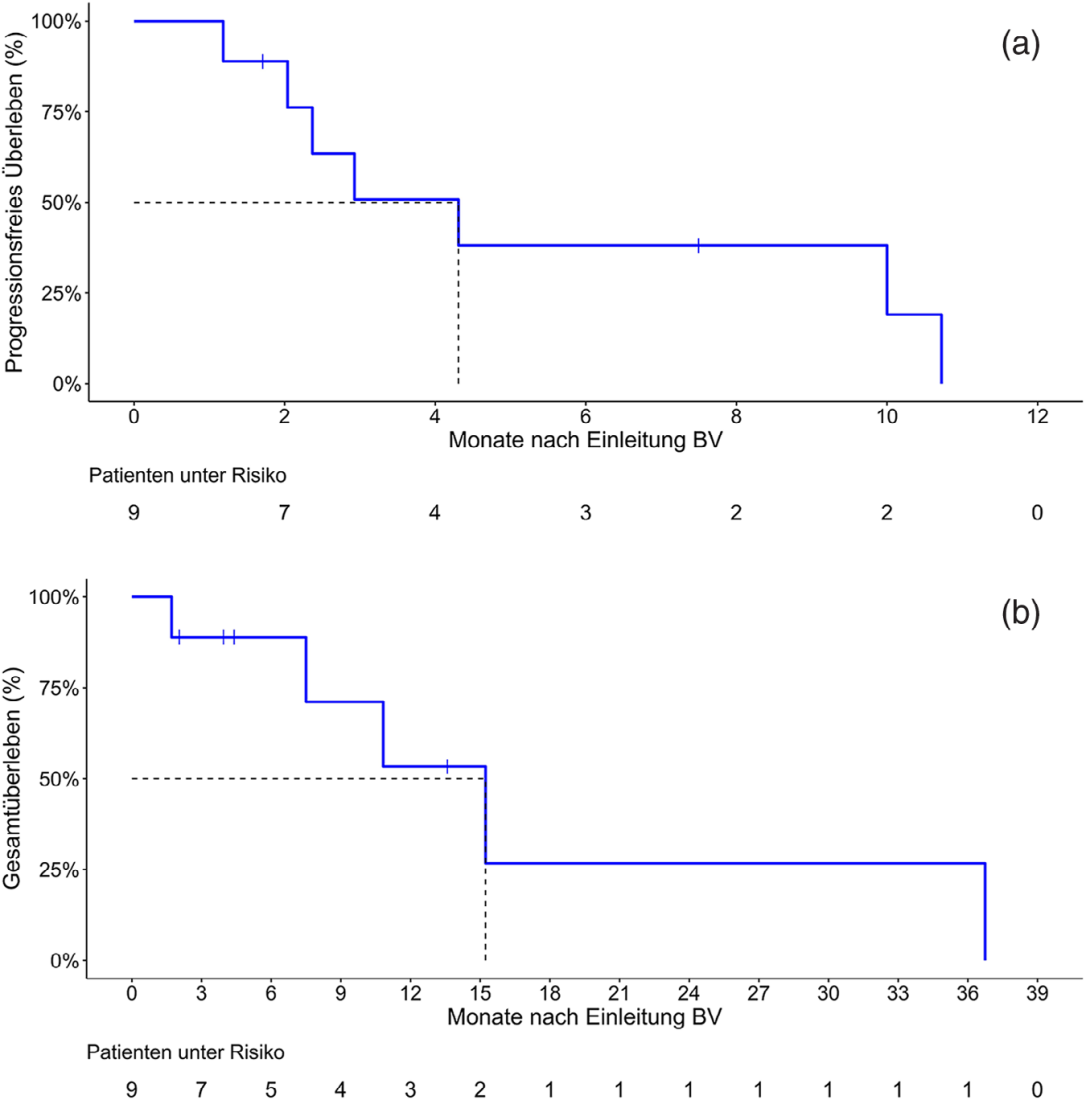
Therapieerfolg nach Behandlung mit BV als Monotherapie oder Kombinationstherapie. (a) Kaplan‐Meier‐Diagramm zum progressionsfreien Überleben der Gesamtkohorte (n  =  9). Medianes progressionsfreies Überleben seit Einleitung der Therapie mit BV als Mono‐ oder Kombinationstherapie: 4,3 Monate (95%‐KI: 2,4–NA). (b) Kaplan‐Meier‐Diagramm zum Gesamtüberleben der Gesamtkohorte (n  =  9). Medianes Gesamtüberleben seit Einleitung der Therapie mit BV als Mono‐ oder Kombinationstherapie: 15,2 Monate (95%‐KI: 7,5–NA).

Die mittlere Zeit bis zum Therapieansprechen (CR/PR) war mit etwa einem Monat (31,5 Tage; Interquartilabstand 12–53 Tage) kurz. Nach einem medianen Beobachtungszeitraum von 13,6 (4,4–nicht angegeben [NA]) Monaten dauerte das PFS nach Einleitung von BV im Median 4,3 (95%‐KI 2,4–NA) Monate an (Abbildung 1a). Das mediane Gesamtüberleben betrug 9,0 (95%‐KI 6,1–NA) Jahre seit der Erstdiagnose und 15,2 (95%‐KI 7,5–NA) Monate seit der Einleitung von BV (Abbildung 1b). Das 1‐Jahres‐Gesamtüberleben seit Beginn der Therapie mit BV betrug 53% (25–100%), nach zwei Jahren lag es bei 27% (6–100%). Zum Zeitpunkt der letzten Datenerhebung waren vier von neun Patienten aufgrund eines Progresses an ihrem Lymphom verstorben. Drei dieser verstorbenen Patienten wiesen einen Krankheitsprogress mit nodaler oder viszeraler Manifestation auf, ein Patient zeigte einen kutanen Progress. Ein weiterer Patient verstarb nach einem kardialen Ereignis.

Eine simultane Radiatio wurde unter fortgeführter BV‐Therapie bei neu aufgetretenen beziehungsweise primär oder sekundär resistenten Hauttumoren durchgeführt. Bei einem Patienten erfolgte eine frühzeitige Bestrahlung nodaler Lymphommanifestationen ergänzend zur BV‐Gabe. Eine Ganzhautbestrahlung (*total skin electron beam radiation*, TSEBT) erhielt keiner der Patienten. Alle Patienten mit kutanem PTCL sprachen auf die Kombinationstherapie mit lokalisierter Radiotherapie an (4/4). Drei von vier Patienten erreichten eine partielle Remission (PR), ein Patient eine nahezu komplette Remission (nearCR) als bestes Ansprechen. Bei vier von neun Erkrankten wurde an die BV‐Therapie eine Folgetherapie angeschlossen (Tabelle 2); ein Patient mit PR unter BV erhielt anschließend eine allogene Stammzelltransplantation mit einer Überlebenszeit seit Therapieeinleitung von BV von 21 Monaten.

Während der Therapie mit BV traten bei sieben von neun Patienten unerwünschte, für BV bekannte Nebenwirkungen jeglichen Schweregrads gemäß CTCAE auf (periphere Polyneuropathie [PNP]: n  =  6/9; Hämatotoxizität: n  =  5/9) (Tabelle 3). Schwere Nebenwirkungen (≥ Grad 3 nach CTCAE) wurden bei vier von neun Patienten beobachtet (Neutropenie, Lymphopenie, PNP). Die Therapie musste bei keinem Patienten aufgrund von Nebenwirkungen vorzeitig abgebrochen werden. Eine Patientin entwickelte unter kombinierter Therapie mit BV und simultaner lokalisierter Radiatio bei hoher kutaner und nodaler Tumorlast ein Tumorlysesyndrom. Todesfälle infolge von Nebenwirkungen traten nicht auf. Es fanden sich keine Hinweise auf ungewöhnliche oder unerwartete Toxizitäten oder eine Potenzierung von Nebenwirkungen durch die Kombinationstherapie.

**TABELLE 3:** Unerwünschte Wirkungen während der Therapie mit BV.

**Unerwünschte Wirkungen (UAW)**	**Patienten n/n (%)**
Jeder Schweregrad	7/9 (78)
> 1 UAW	5/9 (56)
≥ Grad 3	4/9 (44)
**Organsystembeteiligung**	
Neurologisch (Polyneuropathie)	6/9 (67)
Hämatologisch	5/9 (56)
Leber/Pankreas	2/9 (22)
Niere	1/9 (11)
Andere^*^	3/9 (33)

Aufgrund von Rundungen kann die Summe der Prozentsätze von 100% abweichen.

* Andere dokumentierte UAW umfassen: Soorösophagitis, Strahlendermatitis, Fatigue, Gewichtsverlust, SARS‐CoV‐2‐Infektion, Ulzeration der Tumorknoten.

## DISKUSSION

Die Behandlung des kutanen PTCL stellt aufgrund des häufig aggressiven Verlaufs mit hohem Risiko für eine systemische Disseminierung und entsprechend hoher Letalität eine therapeutische Herausforderung dar.^11^, ^12^ Bevorzugt eingesetzte (Poly‐)Chemotherapien zeigen bei PTCL meist ein kurzes Ansprechen mit Rezidiven innerhalb weniger Monate.^2^, ^11^, ^13^ Die Therapie eines Patienten mit kutanem PTCL bleibt häufig eine Einzelfallentscheidung.^3^ Randomisierte Studien zum Einsatz von Brentuximab‐Vedotin bei kutanem PTCL wurden bislang nicht durchgeführt. In dieser retrospektiven Erhebung evaluierten wir deshalb BV als Mono‐ oder Kombinationstherapie bei Patienten mit CD30‐positiven PTCL (n  =  9) hinsichtlich Therapieschema, Verträglichkeit und Effektivität.

BV erwies sich in unserer Kohorte als Mono‐ oder Kombinationstherapie als rasch wirksam und gut verträglich. Im hier dargestellten Patientenkollektiv, in dem BV als Zweit‐, teilweise auch als Dritt‐ oder Spätlinientherapie eingesetzt wurde, wurde im Mittel bereits nach 31,5 Tagen ein Therapieansprechen beobachtet. Insbesondere vor dem Hintergrund der raschen Krankheitsprogression ist diese Beobachtung von Bedeutung, da eine zeitnahe Diagnosestellung, ein früher Therapiebeginn und ein schnelles Ansprechen die Prognose verbessern können.^3^

BV wies weder als Mono‐ noch als Kombinationstherapie bei Patienten mit PTCL ein ungewöhnliches Nebenwirkungsprofil auf.^7^ Die beobachteten Nebenwirkungen entsprachen denen früherer Studien.^7^, ^11^ Eine periphere Polyneuropathie trat bei 67% der Patienten unserer Analyse auf, verglichen mit 69% in der ALCANZA‐Studie. Schwere Nebenwirkungen (≥ Grad 3 nach CTCAE) wurden bei 44% der Patienten in unserer Kohorte beobachtet, gegenüber 41% in der ALCANZA‐Studie.^7^

Die gute Verträglichkeit von BV sowohl in der Mono‐ als auch in der Kombinationstherapie sowie die Tatsache, dass die häufigsten Nebenwirkungen gut kontrollierbar waren und in unserer Fallserie keine Therapie aufgrund von unerwünschten Nebenwirkungen beendet werden musste, erlauben auch zukünftig den Einsatz von BV bei kutanen PTCL.

Das Ansprechen auf BV war bei der Mehrzahl der Patienten dieser Analyse initial gut: Sechs von neun Patienten erreichten eine CR oder PR, was der objektiven Ansprechrate (CR und PR; ORR) der ALCANZA‐Studie entspricht (BV‐Arm: 65,6%; Kontrollarm: 20,3%).^8^

Patienten mit einer CD30‐Expression ≤ 10% in der ALCANZA‐Studie zeigten kein schlechteres Therapieansprechen auf BV als Patienten mit einer höheren CD30‐Expression.^14^ Eine höhere CD30‐Expression führte auch in unserer Analyse nicht notwendigerweise zu einem besseren oder längeren Ansprechen.

Trotz der schnellen Wirkung, des guten Ansprechens und der guten Verträglichkeit von BV bei PTCL hatten die Patienten unserer Analyse eine schlechte Prognose. Mit einem medianen PFS von 4,3 Monaten war die Ansprechdauer bei PTCL begrenzt (Abbildung 1a). Im Gegensatz dazu erreichten MF und pcALCL Patienten in der ALCANZA‐Studie unter BV ein fast viermal längeres medianes PFS (16,7 Monaten).^7^

Der Großteil der Patienten in unserer Analyse wies ein fortgeschrittenes Krankheitsstadium mit generalisierter Hautbeteiligung (T3 nach EORTC/ISCL) auf. Auch in der Literatur ist dieser Befund mit einer signifikant schlechteren Prognose assoziiert als lokalisierte Verläufe (p < 0,034).^2^, ^3^, ^11^

Die derzeit verfügbaren Therapieoptionen für Patienten mit PTCL zeigen – ebenso wie Brentuximab‐Vedotin – nur eine begrenzte Langzeitwirksamkeit.^1^ Die Hälfte der Patienten überlebte nach Einleitung der BV‐Therapie weniger als 15 Monate. Aufgrund des raschen und guten Ansprechens käme BV jedoch als remissionsinduzierende Therapie vor einer Stammzelltransplantation potenziell infrage.^15^ Das bestätigt auch ein Patient dieser Analyse, der mit BV eine PR erreichte und anschließend eine allogene Stammzelltransplantation erhielt. Seit Therapieeinleitung bis zum letzten Follow‐up lag sein Überleben bei 21 Monaten.

Die Effektivität von BV bei kutanen PTCL im Hinblick auf das progressionsfreie und das Gesamtüberleben sollte in prospektiven, randomisierten klinischen Studien weiter untersucht werden – sowohl im Rahmen einer optimierten Kombinationstherapie als auch als remissionsinduzierende Behandlungsoption vor einer Stammzelltransplantation. Daher ist es unseres Erachtens essenziell, klinische Fälle von Patienten mit kutanen PTCL in Registern zusammenzutragen, um die Versorgung der Patienten, trotz aktuell limitierter Therapieoptionen, langfristig verbessern zu können. Zudem besteht weiterhin ein hoher Bedarf für neue Therapieoptionen bei kutanen PTCL.

## DANKSAGUNG

Caroline Glatzel wurde durch das TWINSIGHT‐Clinician‐Scientist‐Programm (Projektnummer: TWINSIGHT‐09) an der Medizinischen Fakultät der Universität Würzburg unterstützt, das von der Else‐Kröner‐Fresenius‐Stiftung finanziert wird.

Open access Veröffentlichung ermöglicht und organisiert durch Projekt DEAL.

## INTERESSENKONFLIKT

C.G. erhielt außerhalb der eingereichten Arbeit Honorare von Recordati Rare Diseases. I.H. erhielt außerhalb der eingereichten Arbeit Reisekostenzuschüsse von Kyowa Kirin. M.G. erhielt Honorare, sämtlich außerhalb der Thematik dieser Publikation, für Mitarbeit in Advisory Boards beziehungsweise für Vorträge von Almirall, Argenx, Biotest, GSK, Janssen, Leo Pharma, Lilly, Novartis und UCB. M.W. erhielt außerhalb der eingereichten Arbeit Honorare und Reisekostenzuschüsse von TAKEDA, Recordati Rare Diseases, Stemline Therapeutics und Kyowa Kirin. Alle anderen Autoren erklären, dass kein Interessenkonflikt besteht.
